# Essential Nature of Caries. No. 3

**Published:** 1867-11

**Authors:** H. R. Noel

**Affiliations:** Professor of Physiology, Baltimore College of Dental Surgery.


### AMERICAN JOURNAL

OF

### DENTAL SCIENCE.

Vol. I. THIRD SERIES--NOVEMBER, 1867. No. 7.

### ORIGINAL COMMUNICATIONS.

### ARTICLE I.

### *Essential Nature of Caries. No.* 3.

(Continued.)

By H. R. Noel, M.D., Professor of Physiology, Baltimore College of Dental Surgery.

A Pertinent inquiry suggests itself at once. Does the disease advance now, from the direction of the pulp, and attack the inner surface of these lines of consolidation; or does it advance from without inwardly, and therefore directly through this "zone of consolidation," through this new, and now solid line of dentine just formed? Does it go on towards the pulp, and having removed "the zone" again assail both fibrils and dentine? The quotation from Mr. Tomes answers the question, but practical experience answers it and asserts that the disease advances now as always from exterior to interior, for if otherwise, then would filling be indeed an absurd precaution.

Another fact of importance is this, that in the consolidation of the tubes or rather calcification of the fibrils; in the formation of this "zone of consolidation," this breastwork thrown up for defence, nature has emphatically withdrawn her *vital forces* from the *front* and concentrated *behind this zone.* And significant indeed is this withdrawal.

The nuclei are dead, calcified; the "germinal material" calcified, the whole fibril calcified to the extent of the breadth of this barrier, breastwork or zone, and therefore as incapable of exhibiting vital phenomena *as a piece of iron wire* of the *same length.* But strange to say we are here barricaded out from the only elements of the tooth that are really vitalized; the disease and cause are left outside of this barricade, and the tide of vitality has receded leaving physical forces alone at work. The cause or power operating is an external one here, and the process a purely physical one until the zone is removed, and the fibrils again reached.

Ergo—and we defy any escape from the conclusion, ergo—caries in this case has no element whatever of vitality and is a purely physical or chemical affair. And what is worse still for the vital theory, this is not the exception, but *the rule.* Nature's rule is to barricade out both cause and effect—both agent and disease; both are external and abhorrent to her, and she resists by a systematic barricading.

In fact in many instances, successive lines of these barricades, "zones of consolidation" mark the struggle and mark her retreat to the pulp; taking with her every element of vitality to this the last fortress.

Mr Tomes thinks the *death of the dentine* to be the necessary *prerequisite* to caries; being dead it is acted upon by the cause—but *resists while alive.* This is an assumption of the purest kind—utterly unsupported by one single fact, and in direct opposition to the very phenomena of decay as described by himself. The only vital element that we have so far found as belonging to, or rather as being often present in caries, is the effort by which the zone is produced, and this, strange, very strange to say, is conservative, protective, preventative, and *yet* consists in killing, absolutely and emphatically killing the fibrils to form a barricade against the enemy.

Nature calcifies the nuclei, calcifies the "germinal material," kills the portion of fibril for self-preservation; the death of the dentine therefore, in this case, is the effort of nature to preserve the tooth.

3. Conduction of Sensation by Fibrils.

This is an absolute fact, and the explanation of it is purely conjectural, and has no very practical bearing upon the "Essential Nature of Caries," yet we will give it a thorough investigation.

That the peripheral ends of the fibrils, when exposed and irritated by the air and fluids of the mouth, become very sensitive is a fact familiar to all dentists. That there must be some means of connection between the peripheral end and nerves of the pulp is equally certain; that it can only be along and through the fibril is a necessary and inevitable result of the situation. How the fibrils conduct or transmit sensation, is a matter of no moment to us, the practical fact that they do, is the same; and they are as much isolated as the axis cylinder of any nerve tubule is, as the fibril is surrounded by bony structure. And we believe there is free and uninterrupted passage from the dentinal superficies, along the fibrils to the pulp and its nerves.

We have never accepted the usual explanation given by physiologists, of the terminal distribution of nerve fibres, as it is totally inadequate to explain their varied phenomena. We believe in this *special case*, the nerve fibre by its axis cylinder is continued on in organic continuity with the dentine fibril, losing its distinct individuality as an axis cylinder and blending with the fibril, but retaining in this position, or rather imparting to the fibril, some of its own powers, i. e. reception and transmission of impressions. As a fibril it has the same isolation it possessed as an axis cylinder, and the practical fact, theorize as we may about it, remains; the fibrils are capable of reception and conduction of sensations and impressions. We might say that conduction alone, requires isolation and retention of distinct individuality as an axis cylinder, but that the reception of impressions &c., at the peripheral ends was facilitated by a blending or fusion of the axis cylinder with tissues, surrounding—this fusion one of organic continuity.

The objection might be raised here, that in caries, there is often extreme sensitiveness, and therefore vital phenomena exhibited, hence the fibrils enter again into the discussion. This we candidly acknowledge, and readily explain ; if the zone of consolidation be not complete ; if it be imperfect, &c., we shall of course have sensitiveness ; and the organic changes going on in the fibril after cutting or injuring it, may give rise to extreme sensitiveness ; other agents may do the same, such as action of air, fluids of the mouth, &c. But once this zone thoroughly completed and all sensitiveness disappears, but the disease and cause are still external and may or may not be active. The sensitiveness proves nothing therefore, as regards the "Essential nature of Caries," it only proves that one or more fibrils are involved in this special case. And here in opposition to Mr. Tomes, we assert that pain is not a very common element in decay primarily, and by no means an essential element. When present it is only incidental, and has no practical bearing upon the nature of decay.

Caries in enamel is never marked by pain, and carious cavities may exist in dentine and often do, without occasioning the least inconvenience. We examined carefully the mouths and teeth of ten persons, who asserted that their teeth were perfectly sound, had never been painful or even sensitive ; eight of this number had carious teeth, and some three or four had very decided cavities. Pain therefore is not an essential element of caries, and we exclude it.

We have given and we think, fairly given the whole of the vital phenomena of the fibrils, the whole of their vital endowments ; we have discussed them at some length, but have failed to find any vital element, invariably present in caries ; not one, enters as an essential. Eliminating step by step, the incidental elements present in different cases of caries, we have arrived at last, at that point, whence the essential elements, the ever present, the invariable, rise to their true position—and their true significance is appreciated.

That no mistake may occur, we repeat that as caries can and does occur, without any vital element entering into the process, it cannot be claimed that caries involves other than physical forces.

It is intrinsically other than a vital process. We refer of course to caries of teeth, and not of bone. We believe caries of teeth to be an affection "SUI GENERIS."

We have examined various writers upon caries of bone, and we have *carefully examined* them, and we have utterly failed to establish the analogy between the two; it is often assumed, but no where have we found it substantiated by proof.

Dr. Beale, perhaps one of the best microscopic anatomists alive, has advanced novel ideas as regards caries of bone, but upon caries of teeth he advances no theory, and we are left with a few vague and general surmises. As regards bone—his theory, is to say the least of it, fanciful in the extreme, and cannot for one moment be applied to caries of teeth.

"In caries, the germinal matter (nuclei and nucleoli) of a part of a bone, receives too large a supply of nutrient matter, it grows too fast, and lives upon the surrounding tissue which has been already formed." --*Structure, Growth, and Life. p.* 145.

Here a nutrient fluid is acknowledged to permeate the tissue and caries is described as an affection of the nuclei or germinal centres, and not of the ossified tissue, involving the latter only secondarily. Now hear Dr. Beale upon teeth.

"In dentine, tubes or canals containing air, have resulted from the drying up of the soft matter which occupied them during life, and to these *artificial* channels the office of transmitting nutrient fluid to every part of the tissue has been assigned, but, as it seems to me, upon most insufficient grounds."—*Idem p.* 147.

"There is no evidence of addition and removal of material going on in the enamel and dentine after the completion of their formation, and it is probable, that the matter upon which the hardness of these tissues depends, is not removed at all after its deposition."—*Idem p.* 149.

We make no comment here, as it would be supererogation, in fact it would be "an attempt to paint the lily."

Mr. Tomes is a warm advocate of the "Vital Theory," and he supports his position by arguments from analogy ; changes occurring in bone, in skin, in mucous membranes, in the soft structures generally; ulcerations, abscesses, &c., are all brought in, Now we have but little to say of this manner of arguing, but that little is strong ; 1st. His arguments are weak. 2nd. They are not legitimate. 3rd. They are anything else than conclusive. 4th. By neither arguments or theory can he explain, the very phenomena of decay, which he himself so ably describes. Practical observation forced Mr. Tomes to the wildest theorizing to support his pet theory.

The phenomena in the case of the soft structures and the phenomena in the case of the teeth are so widely different, the structures so different, the amount of vitality so different, that all arguments by analogy from such sources are worthless—perfectly so.

The *assumption* of the death of the dentine before it can be decomposed, involves also the *assumption* of its vitality ; now we have given this point an elaborate and we think, a sufficient discussion, as regards both enamel and dentine; and we unhesitatingly assert that the whole vital theory of caries is founded upon the assumption of an amount of vitality in enamel and dentine, that neither practical experience, nor the latest physiological researches of Dr. Beale, &c., in any sort of manner justify.

Neither physiology nor practical experience sustain the vital theory. Even Dr. Beale, though evidently inclined to gravitate into the vital theory, is unable to do so, having by direct microscopic observation and logical theorizing, hermetically sealed himself outside of the vital, as regards teeth.

One more statement by Mr. Tomes and we shall conclude our article.

(Dental Surgery page 373) Speaking of carious cavities containing an acid, he remarks that if *unresisted* by the *vitality* of the *dentine,* it is capable of decomposing it—i. e. causing caries to extend.

Now this is a most unfortunate and unwarrantable assertion, for the "zone of consolidation" so admirably described by himself, has put a barrier between the vitalized fibrils and the acid, and the acid acts first upon this zone, and this zone according to his own statements, is *protective, restrictive, conservative,* as regards the caries; though as we have most certainly shown, is not *a vitalized structure,* and if perfect consolidation has taken place, no where in the limits of this zone are any vital phenomena whatever possible.

The degree and completeness of consolidation marks two things. (1)—Loss of vitality absolute. (2)—increased power of resistance. This is unquestionably true, but in direct and emphatic opposition to Mr. Tomes' statement.

Now there are certain facts—certain conclusions; certain propositions which we shall place before our readers, and which must inevitably weigh against the vital theory.

I.—The invariably external existence of the cause. Bone, &c., have diseases from internal or vital causes.

II.—Prior to *eruption,* caries never occurs—Mr. Tomes' arguments by analogy from bone are therefore fallacious.

III.—If caries be a vital process—filling teeth as Dr. Arthur ably argues, should increase the predisposition to it, as *dentine* is *more injured and its vitality of course lowered.*

IV. Death of the pulp does not necessitate caries at once, though the *dentine is now surely dead,* and filling of the pulp cavity does not increase the tendency to caries, or modify it in any manner, except as regards pain and the "zone," of course both of them are absent.

V.—Dry and dead teeth, placed in as artificial substitutes, undergo a well defined and characteristic caries, yet here vital agency is of course impossible.

VI.—Decay never *begins* in the dentine, unless the enamel being defective, inadequate protection is offered. Accurately covered by enamel, dentine *never decays.*

VII.—Decay never returns in an accurately and well filled cavity, if all the surrounding tissue be intact. This question is ably discussed in the American Journal of Dental Science, Oct. No., 1851, Article I. by R. Arthur.

VIII.—Cementum is never attacked first, unless the neck of the tooth be denuded.

IX.—Filing teeth, which theoretically and practically removes the enamel, removes a portion of the dentine, tears and lacerates the fibrils, and of course irritates and lowers their vitality, does not intrinsically increase the predisposition to decay, on account of any *injury* done the *dentine.*

X.—On the contrary—practical experience, substantiated by statistical data, demonstrates the fact, that filing the teeth apart and keeping them permanently separated is the true

PROPHYLAXIS.

XI.—That after filing, the first effort of nature is the "zone of consolidation," which is nothing more or less than a calcifying of the fibrils, and a condensation and change of a vitalized into a non-vitalized substance.

XII.—That rapid decay is marked by extreme sensitiveness, pain, &c., which are certainly very inferior proofs of death, but positive evidences of organic change progressing in the fibrils, the only vital element of dentine.

XIII.—That enamel,—that dentine without fibrils,—and the zone of consolidation are non-vitalized structures in the true sense of the word "vitalized," and as decay occurs in these structures, the conclusion is inevitable, and the inexorable, logical deduction remains to us. "That in its essential nature caries has no element of vitality."

We are well aware, that the conclusion arrived at is opposed to the views of eminent men both in America and England, yet we do not for one instant hesitate to assert, that they must either ignore the latest researches in physiology or discard their theory. The issue is made—and Dr. Beale has, unintentionally it is true, but none the less ably, demolished the foundation of the vital theory. *Non-vitalized* structures cannot have *vital diseases.*

The objection to filing, so warmly urged by the vital theorists, as being injurious to the vitality of dentine and therefore predisposing to decay, has no foundation either in fact or theory. Neither the *practical results* of filing, nor scientific physiology sustain for one instant the objection.

Before the touch of rigid analysis, and the light of practical experience, their theory and practice alike fall.

Filing does not by any injury to dentine, predispose to decay; it may incidentally, give dentine instead of dense, firm, compact enamel, to the action of the agent—and dentine is more readily eroded.

But even here we are met and silenced by practical experience, supported by physiology and dental pathology, for the "zone of consolidation" compensates and presents a surface, which is practically as good as enamel.

The destroying agent is external; the process not a vital one but physical or chemical; teeth by approximating, retain the agent longer and more closely in contact than would otherwise occur, hence the rapidity and extent of its action upon approximating surfaces. We therefore conclude that open spaces are indicated, and filing and cutting away portions of dentine as well as enamel; i. e. effecting a permanent separation receives the sanction of sound physiology, as it has that of practical observation.

The denial of vitality, to dentine and enamel, the zone of consolidation following the file, give us the physiological solution of the results of practical experience. Dr. Arthur gave us the *statistical facts*, we have endeavored to give their solution by physiology.

---

### ARTICLE II.

### *What constitutes the Respiratory Changes?*

